# Intraoperative Conversion from Endoscopic to Open Transcortical-Transventricular Removal of Colloid Cysts as a Salvage Procedure

**DOI:** 10.7759/cureus.247

**Published:** 2015-02-02

**Authors:** Joseph A Osorio, Aaron J Clark, Michael Safaee, Matthew C Tate, Manish K Aghi, Andrew Parsa, Michael W. McDermott

**Affiliations:** 1 Department of Neurological Surgery, University of California, San Francisco; 2 Neurological Surgery, Northwestern University Feinberg School of Medicine; 3 Department of Neurosurgery, University of California, San Francisco

**Keywords:** colloid cyst, endoscopic, transcortical, interhemispheric, surgical approach

## Abstract

*Objective: *To describe the transcortical-transventricular as an intraoperative salvage procedure and its effect of operative time and outcome.

*Methods:* Thirty-three patients were included in the study. Twenty patients had an endoscopic operation, five had a transcortical-transventricular approach, and eight underwent an interhemispheric approach for resection. Based on common cyst location in the roof of the third ventricle, we propose a simple classification of surgical operative zones based on relationships defined by the anterior column of the fornix, the septal vein, and the medial atrial vein.

*Results:* Complete capsule removal was achieved in 35% of endoscopic operations, 100% of transcortical-transventricular operations, and 63% of the interhemispheric operations. Operative time was 176 minutes for endoscopic operations, whereas the operative time for cases that converted to the transcortical-transventricular approach was 190 minutes (p=0.39).

*Conclusion:* A surgical-based classification of zones within the roof of the third ventricle that can be accessed with microsurgical techniques is proposed. Both endoscopic and microsurgical cyst aspiration and excision remain options. We believe that younger patients, patients with large cysts that fill the third ventricle, or those with recurrence after prior treatment would benefit from open transcortical excision as a safe and effective operative approach using modern image-guided systems.

Consent was formally obtained or waived for all subjects present within this study.

## Introduction

Colloid cysts are endodermally derived benign intracranial lesions located in the third ventricle [1]. They are relatively rare, accounting for 0.5-1% of brain tumors. Surgical treatment of colloid cysts is aimed at relieving or preventing neurologic decline related to obstructive hydrocephalus. Approximately half of patients will present with symptoms with the remainder being incidental findings [2]. Patients with symptomatic cysts are more likely to be younger, have larger cysts, have increased ventricular size, and have increased signal on T2 sequences [3]. A small percentage of asymptomatic colloid cysts will progress to requiring surgery [2].

When surgical cyst removal is indicated, the choice of surgical approach is controversial. Current recommendations for definitive surgical removal range from an endoscopic approach, to an open craniotomy for transcallosal approach, or to an open craniotomy for a transcortical-transventricular approach [4-10]. Some have described a shorter operative time, lower complication profile, and shorter hospital stay with endoscopic approaches [4]. Others report significantly higher rates of complete cyst removal, including the capsule, with open cranial approaches [11]. A recent large series of 35 consecutive patients treated endoscopically reported that the operation was converted to open craniotomy in six patients due to forniceal adhesion of the cyst [12]. The current literature is primarily comprised of series describing outcomes of the three procedures [5-6, 13-14]. The majority of comparative studies address the endoscopic and transcallosal approaches [11]. Some have combined the transcallosal and transcortical-transventricular groups into a single open craniotomy group [4, 15]. Few have directly compared the endoscopic to the transcortical-transventricular group [16]. No studies have described the transcortical-transventricular as an intraoperative salvage procedure and its effect on operative time and outcome.

## Materials and methods

### Patient population

Over the course of last 10 years (1992 – 2012), a retrospective analysis was performed at our institution to examine all operative resections of third ventricular colloid cysts. Thirty-two patients were included in this study. Tissue samples from all patients were obtained at the time of the operation, and were confirmed by histology to be colloid cysts. Patient ages ranged from 17 to 69 years, with a median age of 37 years. There were 19 men and 13 women included in this study. Patients underwent operative resection either by an interhemispheric approach, an endoscopic approach, endoscopic that was converted to a transcortical-transventricular approach, or purely a transcortical-transventricular approach. The senior author has been doing intracranial endoscopy since 1990 and has considerable endoscopic experience. The Committee of Human Research at our institution (IRB #12-08993) approved this 10-year retrospective analysis.

### Colloid cyst characteristics

Preoperative imaging was used to evaluate colloid cyst size and was available for 31 of 33 cases reviewed. MRI was used to measure cyst size in 27 of 31 patients, and CT was used for measurement in four cases. Hydrocephalus was also evaluated using MRI and CT, and this data was available for all 33 cases reviewed.

### Proposed classification of surgical zones for entry into roof of third ventricle

The anatomy of the third ventricle is well known and reviewed beautifully by Rhoton in his microsurgical anatomy papers [17]. Based on past experience with these cysts in the roof of the third ventricle, we propose a simple classification of surgical operative zones based on relationships defined by the anterior column of the fornix, the septal vein, and the medial atrial vein. This surgical corridor classification scheme was not used to attempt to describe cysts size.

Zone 1

**Figure 1:**
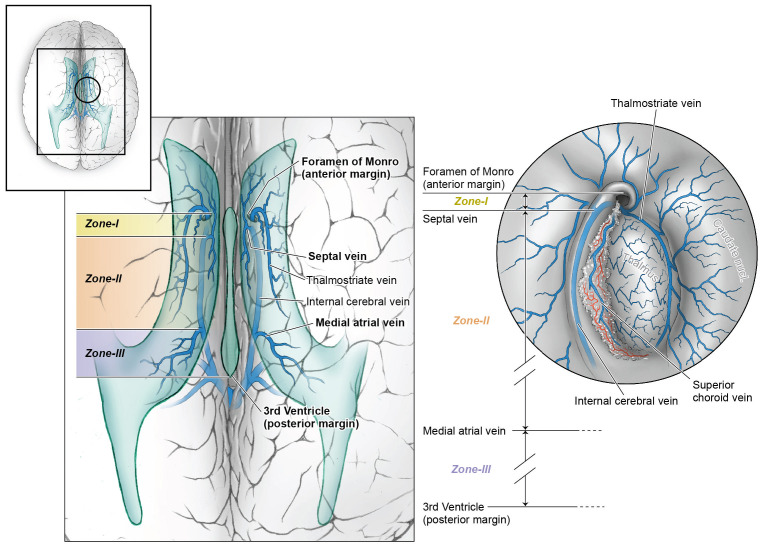
An illustration of the proposed surgical operative zones based on relationships defined by the anterior column of the fornix, the septal vein and the medial atrial vein. Zone 1 extends from the anterior column of the fornix back to the anterior margin of the septal vein.

**Figure 2:**
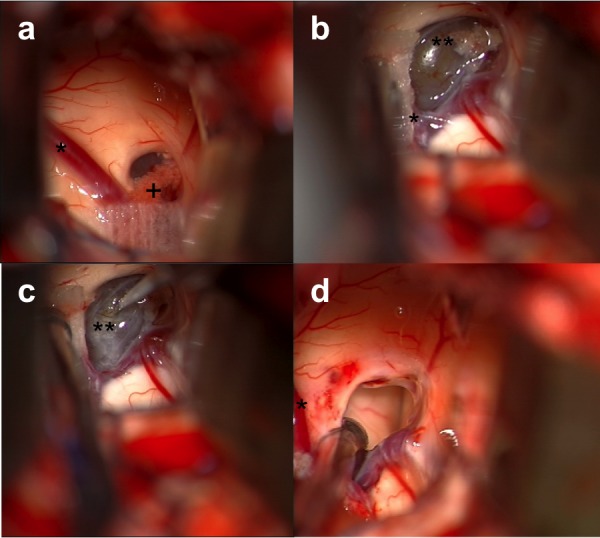
Intraoperative images from transcortical-transventricular approach for Zone 1 cyst. (a) shows initial exposure into ventricle, showing septum pellucidum, Foramen of Monro, choroid plexus (+), and septal vein(*) on left. (b) after coagulation and cutting of choroid plexus; therefore, Foramen of Monro appearing wider, with colloid cyst (**) adjacent to septal, caudate, and thalamostriate veins. (c) incision of wall of colloid cyst (**) with an ophthalmic blade, noting that foramen is kept open with gentle countertraction on column of fornix. (d) aspiration of cyst is shown achieving full excision of cyst wall, and the foramen is open with floor of the third ventricle observed in the distance; the thalamostriate vein shown intact at the 3 o’clock position, and septal vein (*) is shown intact.

Zone 1 extends from the anterior column of the fornix back to the anterior margin of the septal vein (Figure 1). Small colloid cysts can be completely removed in this Zone by opening the tenia choroidea between the fornix medially and the choroid plexus laterally. Without division of the septal vein, a few additional millimeters of exposure of the roof of the third ventricle can be achieved in this way (Figures 1, 2).

Zone 2

**Figure 3:**
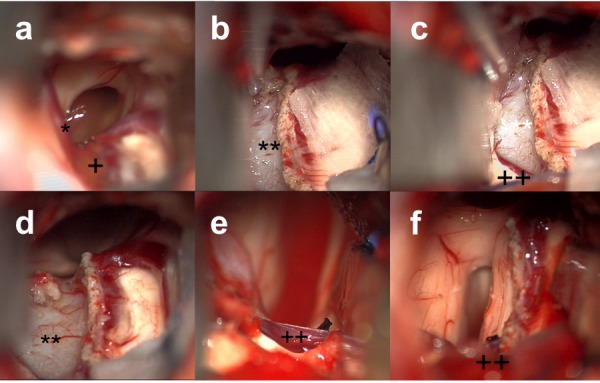
Intraoperative images from transcortical-transventricular approach for Zone 2 cyst. (a) shows initial exposure into ventricle, showing Foramen of Monro, choroid plexus (+), and septal vein (*) on left. (b) after coagulation and cutting of choroid plexus and extension past septal vein; therefore, Foramen of Monro appears wider, with colloid cyst (**) adjacent to thalamostriate vein. (c) Zone 2 dissection is extended to anterior margin of the medial atrial vein (++) that is left intact, colloid cyst (**) is noted encompassing all of Zone 2. (d) colloid cyst (**) is carefully isolated from its adjacent blood supply. (e) medial atrial vein (++) is preserved, and (f) wide exposure obtained after careful resection.

Zone 2 extends from the anterior margin of the septal vein to the anterior margin of the medial atrial vein. Access to this zone requires isolation, coagulation, and division of the septal vein, preserving the patency of the thalamostriate and internal septal veins. Once the vein is coagulated, the tenia fornicea can be opened along the roof of the third ventricle, lateral to the body of the fornix and medial to the choroid plexus and internal cerebral veins. Large cysts in this zone can be carefully isolated from their blood supply from the posterior medial choroidal arteries, and the cyst wall can be excised completely (Figures 1, 3).

Zone 3

Zone 3 extends from the medial atrial vein back to the posterior third ventricle. The medial atrial vein is the posterior limit of the dissection in the roof of the third ventricle, but the aqueduct and posterior third ventricle can be easily seen Figure 1.

### Transcortical-transventricular surgical technique

The patient is positioned supine on the operating table, with the head placed in a Mayfield head pinned apparatus. The neck is flexed on the chest while maintaining a neutral position without rotation. A right frontoparietal coronal scalp incision is made followed by a small mini-craniotomy about 2.5 cm lateral to midline. The dura is opened in a cruciate manner, and the individual leaves of the dura are tacked up peripherally with interrupted 4-0 sutures. The middle frontal gyrus should be underlying the exposure sight. Subsequently, a pial incision is made for introduction of an image-guided rubber catheter into the ventricle. A rubber catheter is initially introduced transcortically in order to guide and identify the location of the ventricular system. After the ventricular system has been identified, the operative microscope is brought into the field and a small cerebrotomy is made in a linear fashion along the middle frontal gyrus. The subcortical dissection is done using the Rhoton #4 microdissector, avoiding use of bipolar or suction which creates a more pronounced tract on postoperative MRI scans and may promote subdural hygromas. Subcortical dissection is continued along the rubber catheter, which serves as a guide during the dissection into the ventricular system. The ependymal surface is lastly identified and opened creating an exposure into the ventricular system. A Greenberg self-retaining retractor system is used for retraction using two retractor blades, one medially and one laterally, to provide the corridor for exposure of the ventricular system.

Once working within the ventricular system, the choroid plexus is identified and is followed anteriorly, as shown in Figure 2. Using a fenestrated suction, the fornix column can be displaced medially, and the choroid plexus laterally to identify the foramen of Monro which will be filled by cyst wall. The choroid plexus is followed and used to identify the caudate and thalamostriate vein. In order to develop a plane to the colloid cyst, often the choroid plexus immediately anterior to the thalamostriate vein travelling over top of the cyst wall is coagulated and divided with straight microscissors.

Cyst decompression is often performed first so that the cyst capsule can be excised safely. To decompress the cyst, first the mid-portion of the capsule is coagulated, followed by a small incision. The contents of the cyst are then aspirated using a non-fenestrated suction, allowing the cyst to collapse. Collapse of the cyst creates a plane around the capsule that allows for small vessels attached to the capsule of the colloid cyst to be coagulated with the bipolar and divided with straight microscissors in Zone 1. For larger cysts in Zone 2, further dissection is required to divide small vessels from the roof of the third ventricle supplying the capsule as shown in Figure 3.

After the colloid cyst has been resected, a small septostomy is created superior and anterior to the foramen of Monro. Lastly, the ventricular cavity is inspected for any bleeding, and this is followed by irrigation with lactate ringer solution. An external ventricular drain is placed into the frontal horn of the lateral ventricle and is brought out through the cerebrotomy wound. The dura is then closed, and the bone flap is repositioned and secured, followed by galeal and skin closure.

### Endoscopy and conversion to transcortical transventricular

Endoscopic procedures were performed with image-guided insertion of a peel-away sheath followed by insertion of the endoscope. Pneumatic holding arms for the endoscope were not used. The surgical team was divided into a navigator for holding and manipulating the endoscope, and a separate surgeon for manipulating instruments for working down one channel. In several cases, a secondary smaller peel-away sheath was inserted anteriorly to allow a secondary working channel for insertion of a grasping or cutting instrument. This was not used on a routine basis (N = 3).

Once the transcortical-transventricular approach was established using the peel-away sheath for the endoscope, the set-up using a mini-craniotomy for the endoscope provided the same exposure needed for a salvage microsurgical approach. When an operation was started using an endoscopic approach, if the surgeon felt that there was an inability to completely excise the cyst capsule after more than an hour of effort, a decision at that point was made to convert to a transcortical-transventricular microsurgical approach. When converting from an endoscopic approach to the transcortical-transventricular approach, the peel away sheath used for the endoscope was removed. The corticectomy was enlarged, self-retaining retractors were used, and the transcortical-transventricular approach followed as described above.

### Operative complications

Complications that were included in our postoperative evaluation were infection, seizure, CSF leak, and a stroke that was identified on diffusion MRI along with clinical symptoms that correlated to MRI changes.

### Surgical outcomes

The surgeon’s operative report was used to evaluate whether the cyst capsule was removed during the case. Postoperative imaging was then used to evaluate radiographic residual. Operative time was calculated in minutes from the time the first incision was made and ending in skin closure. External ventricular drains (EVD) were placed in most cases, and the EVD duration of drainage was measured in days. Length of stay (LOS) was calculated as the time from the operation to discharge from the hospital.

### Statistical analysis

Categorical variables were analyzed by Chi-square test. If any cell contained less than five values, Fisher’s exact test was used. Continuous variables were tested with analysis of variance (ANOVA). Kaplan-Meier was used to generate time to recurrence estimates. Differences were compared by the log-rank test. Analyses were carried out using SPSS version 16.0 (SPSS, Inc.).

## Results

### Patient demographics

Of the 33 patients included in the study, 20 had an endoscopic operation, five had transcortical-transventricular, and eight underwent an interhemispheric approach for resection. The mean age in the endoscopic group was 37, in the transcortical-transventricular approach the mean age was 42, and the mean age of the interhemispheric approach was 40. All patient demographics are shown in Table 1.

**Table 1:** Patient demographics shown using the initial planned operative approach. * The endoscopic column includes both cases that were purely endoscopic cases from start to finish, and cases that were ultimately converted to TC/TV.

	*Endoscopic & Endo -> TC/TV	TC/TV	Interhemispheric	P-value
Sex				
Male	12 (60%)	5 (100%)	3 (56%)	0.08
Female	8 (40%)	0 (0%)	5 (63%)	
Age				
Mean (years)	37	42	40	0.81
Size				
Mean (mm)	12.9	14.6	14.6	0.76

**Figure 4:**
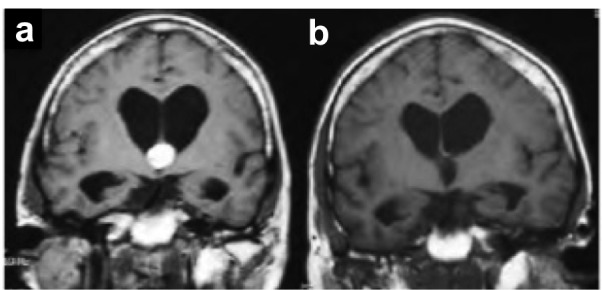
Pre- and postoperative MRIs following transcortical-transventricular resection of a colloid cyst.

Hydrocephalus accounted for 85% of the endoscopic group, 63% of the transcortical-transventricular group, and 80% of the interhemispheric group. Cyst sizes were measured on preoperative MRI or CT; an example of a preoperative MRI is shown in Figure 4.

Patient age, sex, cyst size, and hydrocephalus were not statistically significant between the groups that were analyzed (Table 1).

### Comparison of outcomes between the three surgical groups  

Table 2 provides outcome data between the endoscopic, transcortical-transventricular, and interhemispheric operations. Complete capsule removal was achieved in 35% of endoscopic operations, 100% of transcortical-transventricular operations, and 63% of the interhemispheric operations. These values were statistically significant (p = 0.03). Postoperative imaging evaluation of colloid cyst residual identified complete resection in 61% of endoscopic, 80% of transcortical-transventricular, and 100% of interhemispheric approaches.

**Table 2:** Outcomes for the endoscopic, transcortical-transventricular (TC/TV), and interhemispheric operative approaches shown using the initial planned operative approach. * The endoscopic column includes both cases that were purely endoscopic cases from start to finish, and cases that were ultimately converted to TC/TV.

	*Endoscopic & Endo -> TC/TV	TC/TV	Interhemispheric	P-value
Capsule removed				
Yes	7 (35%)	5 (100%)	5 (63%)	0.03
No	13 (65%)	0 (0%)	3 (37%)	
Radiographic residual				
Yes	7 (39%)	1 (20%)	0 (0%)	0.17
No	11(61%)	4 (80%)	6 (100%)	
OR time				
Mean (min)	183	205	311	<0.001
Mean (min)	183	205		0.7
Mean (min)	183		311	<0.001
Mean (min)		205	311	0.01
EVD				
Mean (days)	1.95	3.2	0.35	0.003
Mean (days)	1.95	3.2		0.14
Mean (days)	1.95		0.35	0.03
Mean (days)		3.2	0.35	0.002
LOS				
Mean (days)	3.5	5.8	5.0	0.04
Mean (days)	3.5	5.8		0.07
Mean (days)	3.5		5.0	0.18
Mean (days)		5.8	5.0	0.7

The mean operative time for endoscopic was 183 minutes, for transcortical-transventricular the duration was 205 minutes, and interhemispheric approach duration was 311 minutes. There was no statistical difference in operative time between the endoscopic and transcortical-transventricular approach (p = 0.7). There was statistical difference between endoscopic and interhemispheric approaches (p < 0.001), and between transcortical-transventricular and interhemispheric approaches (p = 0.01). These values are shown in Table 2.

The average duration (days) for external ventricular drainage (EVD) was 1.95 for endoscopic, 3.2 for transcortical-transventricular, and 0.35 days interhemispheric approach. There was no statistical difference in EVD duration between the endoscopic and transcortical-transventricular approach (p = 0.7). There was statistical difference in EVD duration between endoscopic and interhemispheric approaches (p = 0.03), and between transcortical-transventricular and interhemispheric approaches (p = 0.002). These values are shown in Table 2.

The length of stay for endoscopic was 3.5 days, for transcortical-transventricular was 5.8 days, and interhemispheric approach was five days.

Table 3 compares complications between the various operative approaches. There were no complications observed in the endoscopic and transcortical-transventricular groups, but there were two complications noted in the interhemispheric group. There was one wound infection and one stroke noted in the interhemispheric group.

**Table 3:** Operative complications shown using the initial planned operative approach. * The endoscopic column includes both cases that were purely endoscopic cases from start to finish, and cases that were ultimately converted to TC/TV.

Complications	*Endoscopic & Endo -> TC/TV	TC/TV	Interhemispheric	P-value
Yes	0 (0%)	0 (0%)	2 (25%)	0.04
No	20 (100%)	5 (100%)	6 (75%)	

**Figure 5:**
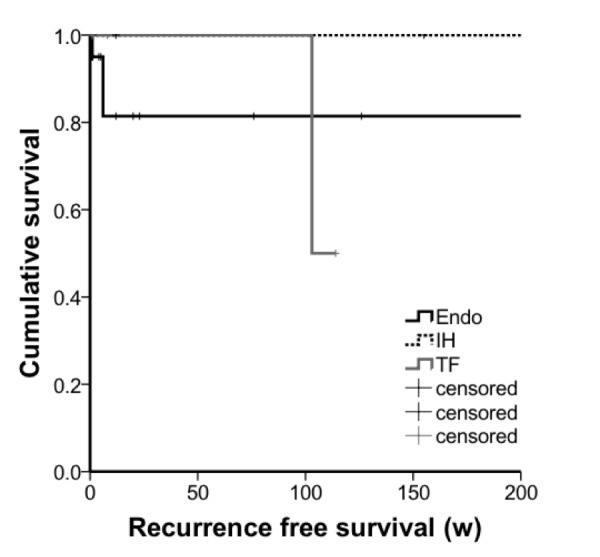
Kaplan-Meier curves used to generate time to recurrence estimates.

Median length of follow-up was five weeks. There were four recurrences; two in the endoscopic group, one in the transfrontal group, and one in the interhemispheric group. There was no difference in recurrence-free survival times between the three surgical approach groups (Figure 5) (p = 0.41, log-rank test).

### Direct comparison between purely endoscopic and endoscopic with conversion to open (transcortical-transventricular)

Table 4 provides a comparison between endoscopic operations compared to endoscopic operations that were converted to transcortical-transventricular. Complete capsule removal was identified in 0% of endoscopic operations, but for those that converted from endoscopic to a transcortical-transventricular approach, 78% identified complete capsule removal. This comparison was statistically significant (p <0.001). Postoperative imaging evaluation of colloid cyst residual identified complete resection in 50% of endoscopic, and 75% of those cases that converted from endoscopic to the transcortical-transventricular approach (p = 0.28).

**Table 4:** Purely endoscopic compared to open conversion: endoscopic converted to transcortical-transventricular (TC/TV).

	Endoscopic	Endo->TC/TV	P-value
Capsule removed			
Yes	0 (0%)	7 (78%)	<0.001
No	11 (100%)	2 (22%)	
Radiographic residual			
Yes	5 (50%)	2 (25%)	0.28
No	5 (50%)	6 (75%)	
OR time			
Mean (min)	176	190	0.39
EVD			
Mean (days)	1.8	2	0.66
LOS			
Mean (days)	3.5	3.4	0.99

Operative time was 176 minutes for endoscopic operations, whereas the operative time for cases that converted to the transcortical-transventricular approach was 190 minutes (p =0.39), shown in Table 4. EVD duration did not vary between these two groups, 1.8 days for endoscopic, and two days for endoscopic converted to transcortical-transventricular (p = 0.66). LOS was not significant between these groups, 3.5 days for endoscopic, and 3.4 days for converted transcortical-transventricular cases (p = 0.99). The comparison between operations that were completed without conversion onto a separate type of operation is shown in Table 5, between endoscopic, transcortical-transventricular, and interhemispheric.

**Table 5:** Purely endoscopic, transcortical-transventricular (TC/TV), and interhemispheric operative outcomes.

	Endoscopic	TC/TV	Interhemispheric	P-value
Capsule removed				
Yes	0 (0%)	5 (100%)	5 (63%)	0.03
No	11 (100%)	0 (0%)	3 (37%)	
Radiographic residual				
Yes	5 (50%)	1 (20%)	0 (0%)	0.17
No	5 (50%)	4 (80%)	6 (100%)	
OR time				
Mean (min)	176	205	311	0.001
EVD				
Mean (days)	1.8	3.2	0.35	0.003
LOS				
Mean (days)	3.5	5.8	5.0	0.04

## Discussion

Pollock, et al. have described the natural history of asymptomatic colloid cysts which indicates that they follow a benign course [2]. Over 25 years, 168 patients with colloid cysts were reviewed, 68 of which were asymptomatic. Of these, 58 were followed for a mean of 79 months. There were no cases of sudden death and the likelihood that asymptomatic cysts would become symptomatic was 0%, 0% and 8% at two, five, and eight years. In a follow-up paper, the same group found that younger age, increased ventricular size, larger cyst size, and increased T2 signal on MRI imaging were associated with a greater likelihood of the cyst becoming symptomatic [3].

Based on modern studies, open microscopic removal of colloid cysts, either by transcortical or transcallosal approach, is associated with a very high rate of complete removal, including the cyst capsule that approaches 100% [6, 11, 15]. Traditionally, it is has been taught that the transcortical approach is associated with a higher rate of seizures than the transcallosal approach. Milligan and Meyer found the opposite when they reviewed the Mayo Clinic experience with surgical approaches to lesions around the third ventricle. The transcallosal approach carried a 4.4 fold increased risk of seizures in the postoperative period compared to the transcortical approach. Hassaneen, et al., in reporting the morbidity of transcallosal approaches to tumors of the third ventricle, found that 34% of patients had a neurologic complication and that sacrifice of parasagittal veins was a factor associated with the risk of complications. Of course, in the transcortical approach, sacrifice of veins is not necessary.

We report similar rates of complete cyst capsule removal as in other microsurgical series. In contrast, endoscopic removal is associated with a lower rate of complete removal, ranging from 10-83% [4-5, 11, 13, 16, 18-19]. Grondin, et al. compared their endoscopic and microsurgical experience for colloid cysts. Complete resection of the cyst capsule was achieved in 12% of the endoscopic group and 100% of the microsurgical group (P < 0.001). The complication rate was higher in the microsurgical group, 32.2% vs. 8.3% (P < 0.001) but the recurrence rate was lower; 0.6% vs. 3.3% (P < 0.003). Horn, et al. comparing the endoscopic to the transcallosal microsurgical approach found that there was no residual on postoperative scans is 53% of endoscopic and 94% of transcallosal cases. In a publication of the Italian cooperative study of endoscopic colloid cyst removal in 11 centers reporting on 61 patients, the planned technique was coagulation of capsule and cyst aspiration. Capsule excision was achieved in 9.8% and the recurrence rate was 11.4%. Recently, Hoffman, et al. reported on the significance of cyst remnants after endoscopic colloid cyst resection, reporting their experience and summarizing the literature [19]. Across all studies of endoscopic treatment, the recurrence rate was 6.3% with a mean time to recurrence of 51 months and a morbidity rate of 13.9%. In the hands of very experienced endoscopic neurosurgeons, the rate of capsule excision may approach that of open cranial operations [12, 20]. A supraorbital endoscopic modification has been proposed to improve visualization of the likely area of cyst adhesion to the third ventricular wall; however, this was not associated with improved rates of complete removal [21]. Others have used frame-based or frameless stereotaxy to place a tubular retractor transfrontally for microscopic removal of colloid cysts [22-24]. Although associated with high rates of complete cyst removal, some have noted that it may be difficult with larger cysts [22]. These alternatives were not compared in the present study. 

While Horn, et al. noted a low rate of complete cyst removal with endoscopy compared to transcallosal approaches [11], they also demonstrate significantly lower operating room time and hospital stay and, therefore, propose that endoscopic removal is appropriate to attempt as the first approach to remove a colloid cyst. If the cyst recurs, then open surgery should be attempted. In the large series by Greenlee, et al, six out of 35 attempted endoscopic colloid cyst operations were converted into open mini-craniotomy [12]. One was due to equipment malfunction, but the others were due to forniceal adhesion and the need for improved visualization. All cases were associated with complete removal suggesting that transfrontal open cranial approaches are efficacious as intraoperative salvage procedures. The effect of conversion to open surgery on the procedure and outcome were not directly compared.

The goal of complete cyst removal including the capsule is to prevent recurrence.  However, this has been conclusively proven as many operations described in case series with subtotal removal do not result in recurrence. Nevertheless, Grondin, et al performed a systematic review of the literature comparing open cranial and endoscopic approaches and reported a statistically significant increase in recurrence with endoscopic approaches (3% vs 0.6%) [4]. This suggests that complete removal may be optimal treatment for these lesions. Our approach to patients with colloid cysts based on this experience is summarized in Table 6.

**Table 6:** Recommendations for Surgical Treatment Based on Colloid Cysts Size and Patient Age. (*) Size: Zone 1 = smaller, Zone 2,3 = larger (+) Options: Endo = Endoscopic removal; TC/TV = Transcortical-Transventricular

Age	Surgical Zone (Size) *	Treatment Option(s)+
Younger	1	Endo or TC/TV
	2,3	TC/TV
Older	1	Endo
	2,3	Endo or TC/TV

This manuscript demonstrates that both microscopic (transcortical-transventricular) and endoscopic approaches remain options for removal of colloid cysts or recurrence of colloid cysts after prior treatment. It also shows that the transcortical-transventricular approach provides an operative approach that is safe and effective with higher rates of complete capsule excision in our hands. No patient in this group had a postoperative seizure. In one case of a left-sided endoscopic approach that took several hours to complete, the patient had a postoperative supplementary motor syndrome that resolved over the next two days. All transcortical-transventricular microsurgical approaches were done from the right.

The classification of surgical zones presented in this manuscript was derived to take into account the surgical approach relative to the dissection along the supra-choroidal corridor, and whether or not the septal vein was divided. Our attempt is not to use this classification for describing the preoperative size of colloid cysts. This classification was based on microsurgical corridors that could be opened with or without dissection, coagulation, and division of the septal vein and the supra-choroidal tenia fornicea, giving wide access to the roof of the third ventricle for the largest cysts. The medial atrial vein provides an anatomical boundary for an approach into the posterior region of the third ventrcle. The medial atrial vein is a large and critical vein, and if sacrificed, it would increase the risk of a thalamic venous infarction, which would be detrimental to the patient. However, Zone 3 can be visualized with changing the angle of the microscope, and with gentle retraction of the capsule of the cyst wall, vascular attachments to the roof of the third in Zone 3 can be coagulated and divided. Thus, cysts that are within the third ventricle under the choroidal fissure of Zone 3 can be accessed looking posteriorly from Zone 2.

There were several aspects of this study that could contribute to limitations when interpreting the data presented. The study was a retrospective analysis that included colloid resection rates that were based on postoperative imaging and intraoperative surgical assessment. The operative reports by the surgeon were primarily used for determining these assessments since postoperative MRI imaging cannot determine whether there is any cyst wall remnant. Also, endoscopic length of stay could be longer in our series because it was routine for our patients to have an external ventricular drain for one to two days postoperatively. Even when taking these limitations into consideration, our experience has provided us with insight to three valuable surgical approaches for colloid cyst resection; we have provided a comprehensive table showing the pros and cons to the different operative techniques presented in this manuscript (Table 7).

**Table 7:** Pros and cons between endoscopic, transcoritical-transventicular, and interhemispheric approaches.

Surgical Approach	Pros	Cons
Endoscopic	Shorter operative times	Requires two surgeons, limited when working in Zone 2 and 3
Transcortical-transventricular	Single surgeon technique, Short operative times	Larger corticectomy
Interhemispheric	Wider exposure, no corticectomy	Longer operative times, technically more demanding, higher risk of complications

## Conclusions

This report documents a high rate of complete colloid cyst capsule excision with a transcortical transventricular approach. A surgical-based classification of zones within the roof of the third ventricle that can be accessed with microsurgical techniques is proposed. Both endoscopic and microsurgical cyst aspiration and excision remain options. We believe that for younger patients, patients with large cysts that fill the third ventricle, or for those with recurrence after prior treatment, an open transcortical excision is safe and effective using modern image-guided systems.
